# The role of attachment relationship in adolescents’ problem behavior development: a cross-sectional study of Kenyan adolescents in Nairobi city

**DOI:** 10.1186/s13034-018-0237-0

**Published:** 2018-06-01

**Authors:** Grace Nduku Wambua, Anne Obondo, Antonia Bifulco, Manasi Kumar

**Affiliations:** 1 Department of Psychiatry, College of Health Sciences, University of Nairobi, P.O. Box 19676, 00202 Nairobi, Kenya; 2 Department of Psychology, Middlesex University, London, UK; 3 Department of Psychiatry, College of Health Sciences, University of Nairobi, 47074, 00100 Nairobi, Kenya; 4 Research Department of Clinical Health and Educational Psychology, University College London, London, WC1E7BT UK

**Keywords:** Insecure attachment, Behavioral problems, Emotional problems, Adolescence, Kenya

## Abstract

**Background:**

There is a significant link between insecure attachment and the development of psychopathology in adolescence. We investigated the relationship between adolescent attachment styles and the development of emotional and behavioral problems among adolescents in Kenya. We also examined the modifying influence of socio-economic-status (SES).

**Method:**

One hundred and thirty-seven adolescents who were attending two schools participated in the study. One school (low SES school) catered for children from predominantly low-income households, while the second school (middle SES school) catered for children from predominantly middle-income households. The data were collected using three instruments: researcher designed questionnaire to obtain socio-demographic information, the Strength and Difficulties Questionnaire (SDQ) that is designed to assess symptoms of disorder, and the Vulnerable Attachment Scale Questionnaire (VASQ) that is designed to measure attachment style.

**Results:**

Adolescents from the low SES school had higher vulnerable attachment scores than those from the middle SES school (*t*(135) = − 2.5, *P *= *0.02*). Male students had higher vulnerable attachment scores than females (*P *= *0.03*). Adolescents who had experienced adversity in childhood had higher vulnerable attachment scores than those who had not (*P *< *0.00*). Results from Pearson’s correlation showed moderate to strong positive correlations between attachment insecurity and emotional and behavioral problems with participants who had higher emotional symptoms (r = 0.47, *P *< 0.01), conduct problem score (r = 0.33, *P *< 0.01), hyperactivity (r = 0.26, *P *< 0.01) and total difficulty scores (r = 0.47, *P *< 0.01), experiencing significantly higher levels of attachment insecurity than those with lower scores.

**Conclusions and recommendations:**

This study supports the notion that attachment insecurity increases the adolescents’ susceptibility to develop psychological problems.

## Background

Mental health problems among adolescents are a significant public health burden in Kenya [1–5]. Increased mental health problems in adolescents compromise their development and future potential [1, 6]. Numerous factors may contribute to adolescents’ mental health problems including poverty related factors, parental marital relations, family disruptions, parental absence, as well as lack of support and cohesiveness in families [7, 8]. However, the influence of poverty and family contextual factors on adolescents’ relationship (i.e., attachment relationship) and mental health functioning is not well understood in Kenya.

Attachment refers to the emotional bond that plays a pivotal role in the regulation of stress in times of distress, anxiety or illness over the course of their infancy [9]. This emotional connection is one of the most important obligations that a parent has to a child. A child who is securely attached is capable of using the attachment figure as a secure base from which they can explore self and the world [10]. The quality and timing of attachment determines the quality of later development. Insecure attachment places the child on a difficult development trajectory throughout life [11].

Adolescence is a period of change of rapid and considerable developmental changes [8, 12]. It is associated with the onset or exacerbation of a number of health-related problems including depression, eating disorders, substance abuse and dependence, risky sexual behavior, antisocial and delinquent activity, and school dropouts [13]. Studies have found attachment security in adolescence exerts precisely the same effect on development as it does in early childhood: *a secure base fosters exploration and the development of cognitive, social and emotional competence* [12, 14]. Adolescents that have a less secure attachment with their parents are more likely to compensate for their emotional disturbances by engaging in problem behaviors [15]. Child rearing practices, parental involvement and parental aggression have been associated with behavioral problems in children [16–18]. These effects become more pronounced as the child gets older.

Psychological studies have demonstrated that the context in which an individual develops is of great importance in understanding and conceptualizing child developmental constructs. Low socio-economic status (SES) and exposure to adverse events are associated with a wide variety of health indicators and psychopathologies. These factors are also linked with variations in parenting and child development [19]. It is important to note here that although many adolescents are exposed to various negative experiences, only a few develop inappropriate behaviors as a consequence [14]. The quality of home life (secure or insecure attachment) significantly prepares the growing child to be more resilient or vulnerable to such influences [17].

In contrast to middle income settlements, informal (or slum) settlements in Kenya are characterized by congestion, high levels of unemployment, inadequate social services, extreme poverty, insecurity, crime, and hopelessness. Comparing adolescents who live in middle-income areas and those living in informal settlements offers a unique opportunity to study how SES may influence the association between attachment security and the development of adolescent emotional and problem behaviors in adolescents [8]. This study was carried out to establish the association between emotional and behavioral problems and adolescent attachment security, and to explore how young participants from differential socioeconomic groups fare vis-à-vis these psychological indicators of attachment security and emotional and behavioral health in Kenya. An extensive body of research exists on the links between attachment security in adolescent and behavioral and psychosocial outcomes later in life, but there is a paucity of research on adolescents’ attachment security and attachment styles in sub-Saharan Africa. This study also serves to bridge that knowledge gap.

## Methodology

### Design and setting

We conducted a cross-sectional study among adolescents attending two secondary schools in Nairobi, Kenya’s capital city. The education system in Kenya caters to both Kenyan and non-Kenyan children. As such, different curriculums are offered including the Kenyan 8-4-4 curriculum, The International General Certificate of Secondary Education (IGCSE or British) curriculum, American curriculum, French curriculum, and German curriculum. Most Kenyan children attend schools teaching the 8-4-4 curriculum.

The schools selected to participate in this study were conveniently chosen by the researchers because they use the 8-4-4 curriculum. The schools were also chosen to represent pupils from different socio-economic classes. A public government funded city-council school (average fees per annum $340) was selected to represent a low-SES school. The school caters primarily to children from neighboring informal settlements. The second school selected is a private school (average fees per annum $2460) located in one of Nairobi County’s middle-upper class suburbs that primarily caters to adolescents from middle income families in Nairobi.

### Participants

We used the cross-sectional design Cochrane formula [20] to determine the minimum required sample size required, using a sample frame of 500 persons. The population proportion of behavioral problems was assumed to be 10% as proposed by Goodman et al. [21], with a confidence level of 95%, and a precision of 5%. A minimum sample size of 138 was computed.

The Kenyan secondary school grades range from Form 1 to Form 4. For this study, we recruited students in Forms 1, 2 and 3. Each form had four classes and the researchers selected two classes from each form to participate in the study. With an average of 25 students per class, every second student was selected to fill out the questionnaires. One hundred and fifty students participated in the study however, 13 questionnaires were excluded in the final analysis because they were incomplete leaving an analytical sample of 137 adolescents ages 14–19 years (*M *= 15.7, *SD *= 1.2). About half (n = 69) of the adolescents were attending the low SES school.

### Instruments

#### Researcher designed socio-demographic questionnaire

Socio-demographic information pertinent to this study was collected via a researcher designed questionnaire. The questionnaire elicited information on the adolescent’s gender and age, the type of school they attend, the caregiver’s relationship to the adolescent; the caregiver’s education level, marital status, and employment status; household income; and household composition. The questionnaire also assessed adolescent’s emotional needs in regard to the caregiver such as parental availability, how the adolescent perceived the relationship with their caregivers. Adolescents also reported on any exposure to, adverse experiences and sexual/physical abuse. These constructs were included to see if there was any relationship or influence with the adolescent’s development of attachment and/or problem behaviors.

#### The strength and difficulties questionnaire (SDQ)

The SDQ is a brief self-report screening tool to detect childhood emotional and behavioral problems that is designed to be completed by children aged 11–17 years [21]. The 25 items in SDQ are divided into five subscales (conduct problems, hyperactivity/inattention, emotional symptoms, peer problems and prosocial behaviors). The statements include; *‘I am restless, I cannot stay still for long’, ‘I am helpful if someone is hurt, upset or feeling ill’, ‘I fight a lot. I can make people do what I want’, ‘I have many fears, I am easily scared’*. The items are scored on a 3-point scale with 0 = not true, 1 = somewhat true, and 2 = certainly true. The subscale scores (range 0–10) are calculated by summing up the scores on relevant items (after recoding reversed items). A higher score on the prosocial subscale reflects strength, whereas higher scores on other subscales indicate difficulties. A total SDQ score is derived from 20 items (emotional symptoms, conduct problems, hyperactivity and peer problem subscale), excluding the prosocial subscale. Scores for total difficulties range from 0 to 40, with higher scores indicating more problems. Participants’ scores can be classified as ‘abnormal/case’ borderline/subclinical and normal utilizing published cut-offs. Reliability of the screening tool can be judged by internal consistency (mean Cronbach α: 0.73), cross-informant correlation (mean: 0.34), or retest stability after 4–6 months (mean: 0.62). SDQ scores above the 90th percentile are associated with an increased probability of independently diagnosed psychiatric disorders [22].

#### Vulnerable attachment style questionnaire (VASQ)

The VASQ is a brief self-report tool that is designed to screen for insecure attachment styles [23]. The VASQ contains 22 short statements, for example; *‘I take my time getting to know people’, ‘I’m clingy with others’, ‘I look forward to spending time on my own’, ‘I feel uneasy when others confide in me’*. On a 5-point Likert scale (5 = strongly agree, 4 = agree, 3 = unsure, 2 = disagree or 1 = strongly disagree), participants rate the extent to which each statement best describes their characteristic style in relation to others. Three scores are computed for the VASQ—the total score, cut off is 57 or higher; level of insecurity/mistrust (avoidant style) sub-score, cut off is 30 or higher; and degree of proximity seeking (anxious style) sub-score, cut-off is 27 or higher. Higher scores indicate a more vulnerable attachment when computing a total score and more insecurity and proximity seeking attachment patterns when using the subscales. Cronbach’s alpha for the overall VASQ and its subscales, insecurity and proximity-seeking, are α = 0.79, α = 0.82 and α = 0.73, respectively [24].

### Procedures

The study was approved by the Kenyatta National Hospital/University of Nairobi Ethics and Research Committee (approval no. P385/06/2014). We received approval from both the Ministry of Education and the National Commission for Science, Technology and Innovation (NACOSTI) to carry out the research. The principals of both schools granted us permission to carry out the research. The aims and procedures of the study were explained to the school administration and teachers in a staff meeting. Informed consent forms that explained the nature and purpose of the study were sent to the parents via the school administration after which assent to participate in the study was sought from adolescents prior to distribution of questionnaires.

The data collection took place in March 2014. As preferred by our participants whose medium of instruction is English, the UK English versions of the questionnaires were used. The researchers helped to explain the meaning of specific words or items in the questionnaires when these were unclear. Adolescents with high SDQ scores were given information about possible emotional and behavioral challenges that their scores indicated and referred to the Kenyatta National Hospital’s Youth Clinic and Department of Mental Health for support. To facilitate follow-up care of adolescents with high SDQ scores, the research team gave the school principals and the school counsellor or liaison teacher a list of pupils with SDQ scores in the abnormally high range.

### Data analysis

Independent sample t-tests, inter-correlation analysis and a hierarchical multiple regression analysis were performed. A correlation matrix was constructed among all the variables, based on Pearson’s co-efficient for significance testing for continuous variables (i.e., problem scores and age). Independent sample t-tests were used to test the association between the selected socio-demographics, emotional needs and experience of adversity among the participants and the attachment insecurity. Variables that were associated with attachment insecurity (P < 0.2) were entered in blocks in the hierarchical multiple regression to test their impact. Prior to the multiple regression analysis, all model assumptions (univariate/multivariate normality, linearity, homoscedasticity and diagnostic testing for multi-collinearity and independence of errors) were tested. Data were analyzed using SPSS version 21, and the level of significance was set at *P *= 0.05, two-tailed.

## Results

### Sample description

Of the 137 adolescents who completed the questionnaire, 47.4% (N = 65) were female (Table 1). Eighty-one percent (N = 111) of adolescents were 14-16 years old. Eighty-five percent (N = 116) of adolescents were living with at least one parent, while 15.3% lived with a guardian. Seventy-five percent (N = 102) of mothers who were living with the adolescents were employed, while 21% were not employed. Seventy-three percent (N = 100) of fathers and 95% (N = 19) of the guardians were employed. Twenty percent (N = 28) of the adolescents reported that they had used drugs before. Twenty-six percent (N = 35) of adolescents had reportedly experienced adversity in childhood, while 5.8% (N = 8) had experienced physical or sexual abuse.

**Table 1:** Socio-demographic factors, emotional needs, experience of adversity among the participants

Parameter	Category	Frequency	Percent
School	Low SES school	69	50.4
	Middle SES school	68	49.6
Gender	Female	65	47.4
	Male	72	52.6
Age	Mean, SD, range	(15.7, 1.2; 14–19)	
Age	14–16 years	111	81.0
	17–19 years	26	19.0
Persons living with	Parents	116	84.7
	Guardian	21	15.3
Religion	Christian	106	77.4
	Muslim	28	20.4
	*Missing*	3	2.2
Parents/guardian marital status	Married	93	67.9
	other	21	15.3
	*Missing*	23	16.8
Mother’s employment status	Employed	102	74.5
	Unemployed	13	9.5
	*Missing*	22	16.1
Father’s employment status	Employed	100	73.0
	Unemployed	5	3.6
	*Missing*	32	23.4
Guardian’s employment status	Employed	19	95.0
	Unemployed	1	5.0
Feels that emotional needs are met by parents	No	46	33.6
	Yes	70	51.1
	*Missing*	21	15.3
Perceived parental relationship	Supportive and loving	76	55.5
	Unsupportive	35	25.5
	*Missing*	26	19.0
Perceived relationship with mother	Available when needed	34	24.8
	Not available when needed	76	55.5
	*Missing*	27	19.7
Perceived relationship with father	Available when needed	61	44.5
	Not available when needed	44	32.1
	*Missing*	32	23.4
Perceived relationship with guardian	Available when needed	8	40.0
	Not available when needed	12	60.0
Drug use	No	109	79.6
	Yes	28	20.4
Experience adversity in childhood	No	76	55.5
	Yes	35	25.5
	*Missing*	26	19.0
Experienced sexual/physical violence	No	123	89.8
	Yes	8	5.8
	*Missing*	6	4.4

The large number of missing values is because these questions are not applicable to those without a guardian

The mean overall VASQ score was 67.1 (*SD* = 8.4), while the mean subscales scores were 35.3 (*SD* = 6.8) and 31.8 (*SD* = 5.9) for the insecurity and degree of proximity seeking scales respectively. Ninety percent (N = 123) of the adolescents had high level of vulnerable attachment. Sixty-one percent (N = 84) were insecure anxious while 16.8% (N = 23) were insecure avoidant.

The mean of the total difficulty scores of the adolescents on the SDQ ranged from 5 to 28 (*M* − 15.8, *SD* = 4.7). The subscales scores ranged from 0 to 19: emotional symptoms (*M* = 3.5, *SD* = 2.4), conduct problems (*M* = 3.0, *SD* = 1.9), hyperactivity (*M* = 5.0, *SD* = 1.4) and peer problems (*M* = 4.3, *SD* = 1.4).

### Socio-demographic factors associated with attachment insecurity

Results from independent t-tests analysis are shown in Table 2. Adolescents from the low SES school had higher vulnerable attachment scores than those from the middle SES school. Male adolescents had higher vulnerable attachment scores than female adolescents. Adolescents with an unemployed mother had higher vulnerable attachment scores than those with an employed mother. Adolescents reporting adverse childhood experiences had higher vulnerable attachment scores than those reporting no adversity. Similarly, adolescents reporting sexual/physical violence had higher vulnerable attachment scores than those reporting no violence. However, the latter association was only marginally significant (P = 0.06).

**Table 2:** Association between attachment insecurity, socio-demographics, emotional needs, experience of adversity

Variable	Category	n	Mean (SD) (VASQ)	Mean difference (95% CI)	Group difference
School	Middle SES school	68	65.4 (8.7)	− 3.49 (− 6.28 to 0.69)	*t*(135) = − 2.5; ***P *= *0.015***
	Low SES school	69	68.9 (7.8)		
Gender	Female	72	65.7 (7.8)	− 3.10 (− 5.91 to 0.29)	*t*(135) = − 2.2; ***P *= *0.031***
	Male	65	68.8 (8.8)		
Age	14–16 Years	111	67.0 (8.0)	− 0.54 (− 4.18 to 3.10)	*t*(135) = − 0.29; *P* = *0.196*
	17–19 Years	26	67.5 (10.1)		
Persons living with	Guardian	21	69.6 (8.3)	2.87 (− 1.06 to 6.81)	*t*(135) = 1.4; *P *= *0.151*
	Parents	116	66.7 (8.4)		
Religion	Muslim	28	65.9 (8.1)	− 1.50 (− 5.08 to 2.09)	t(132) = − 0.8; *P *= *0.410*
	Christian	106	67.4 (8.6)		
Parents/guardian marital status	Married	93	66.8 (8.5)	− 0.26 (− 4.29 to 3.78)	*t*(112) = − 0.1; *P *= *0.900*
	Other	21	67.1 (8.1)		
Mother’s employment status	Employed	102	66.1 (8.4)	− 5.51 (− 10.34 to 0.67)	*t*(113) = − 2.3; ***P *= *0.026***
	Unemployed	13	71.6 (7.6)		
Father’s employment status	Employed	100	66.6 (8.3)	− 1.05 (− 8.51 to 6.41)	*t*(103) = − 0.3; *P *= *0.781*
	Unemployed	5	67.6 (3.5)		
Guardian’s employment status	Employed	19	69.9 (8.6)	5.89 (− 12.72 to 24.51)	*t*(18) = 0.7; *P *= *0.514*
	Unemployed	1	64.0 (7.8)		
Feels that emotional needs are met by parents	No	46	67.9 (9.7)	0.38 (− 2.83 to 3.60)	*t*(114) = 0.2; *P *= *0.813*
	Yes	70	67.5 (7.7)		
Perceived parental relationship	Supportive and loving	76	66.3 (8.4)	− 1.43 (− 4.86 to 2.00)	*t*(109) = − 0.8; *P *= *0.410*
	Unsupportive	35	67.8 (8.6)		
Perceived relationship with mother	Available when needed	34	67.7 (9.7)	1.09 (− 2.40 to 4.58)	*t*(108) = 0.6; *P *= *0.538*
	Never available when needed	76	66.6 (7.9)		
Perceived relationship with father	Available when needed	61	67.4 (8.5)	1.89 (− 1.37 to 5.14)	*t*(103) = 1.2; *P *= *0.253*
	Never available when needed	44	65.5 (8.1)		
Perceived relationship with guardian	Available when needed	8	71.8 (8.5)	3.58 (− 4.61 to 11.77)	*t*(18) = 0.9; *P *= *0.370*
	Never available when needed	12	68.2 (8.6)		
Drug use	No	109	66.8 (7.6)	− 1.71 (− 5.24 to 1.82)	*t*(135) = − 1.0; *P *= *0.339*
	Yes	28	68.5 (11.1)		
Experience adversity in childhood	No	76	65.1 (7.7)	− 6.74 (− 9.96 to 3.52)	*t*(109) = − 4.2; ***P *< *0.001***
	Yes	35	71.9 (8.4)		
Experienced sexual/physical violence	No	123	66.6 (8.5)	− 5.87 (− 11.92 to 0.17)	*t*(129) = − 1.9; *P* = *0.057*
	Yes	8	72.5 (6.8)		

Sample sizes do not add to 137, as there were missing values

Statistically significant (in bolditalic) at the 0.05 probability level

### Correlation between attachment insecurity and emotional and behavioral problems

Results from the correlation analysis (Table 3) revealed moderate to strong positive correlations between attachment insecurity assessed using VASQ and emotional and behavioral problems using SDQ. Adolescents who had higher emotional symptoms (r = 0.47, P < 0.01), conduct problems (r = 0.33, P < 0.01), hyperactivity (r = 0.26, P < 0.01) and total difficulty scores (r = 0.47, P < 0.01) had higher attachment insecurity scores than those with lower scores on these scales. Peer problem score and age were not related to attachment insecurity among the study adolescents (P > 0.05).

**Table 3:** Spearman’s correlation analysis between attachment insecurity and emotional and behavioral problems

Correlations	1	2	3	4	5	6	7	8
1. Total VASQ	1							
2. Level of insecurity	0.712**	1						
3. Degree of proximity seeking	0.604**	− 0.130	1					
4. Emotional symptoms	0.474**	0.359**	0.262**	1				
5. Conduct problem	0.328**	0.292**	0.133	0.311**	1			
6. Hyperactivity	0.258**	0.190*	0.149	0.421**	0.200*	1		
7. Peer problem	0.040	0.039	0.013	0.140	0.077	0.082	1	
8. Total difficulties score	0.469**	0.380**	0.237**	0.812**	0.653**	0.627**	0.424**	1

Italicized numerals are strong positive correlations

** Correlation is significant at the 0.01 level (2-tailed). * Correlation is significant at the 0.05 level (2-tailed)

### Overall model: hierarchical multiple regression

Socio demographic variables (school, gender, age, mother’s employment status), experience of adversity, experience of sexual and/or physical abuse and emotional behavioral problems were entered into the multiple regression model to identify variables that were correlated with attachment insecurity. A hierarchical multiple linear regression model using VASQ total score as the dependent variable and nine predictors in two blocks is presented in Table 4. The overall model with all nine predictors was statistically significant and explained 39.1% of the variance in emotional insecurity. In Block 1, emotional symptoms, conduct problems, and hyperactivity explained 26.4% of the variance in attachment insecurity, which was statistically significant. In Block 2, type of school, gender, age in years, mother’s employment, experience adversity, sexual/physical violence explained 12.7%, of the variance of the attachment insecurity after controlling for emotional symptoms in Block 1. Therefore, the two blocks of variables significantly contributed to the prediction of attachment insecurity.

**Table 4:** Results of hierarchical multiple regression analysis on factors associated with attachment insecurity

Variable	Category	Beta (SE)	95% CI beta	β	t	*P* value	R^2^ change	*F* ratio R^2^ Change
Emotional problems		0.88 (0.38)	(0.13 to 1.64)	0.25	2.3	*0.023*	0.264	10.26**
Conduct problems		0.74 (0.42)	(−0.09 to 1.58)	0.17	1.8	0.080		
Hyperactivity		0.34 (0.58)	(−0.82 to 1.49)	0.06	0.6	0.562		
School	Low SES	2.17 (1.54)	(−0.89 to 5.23)	0.13	1.4	0.163	0.127	2.79**
	Middle SES	*Reference*						
Gender	Male	2.37(1.57)	(−0.76 to 5.49)	0.14	1.5	0.136		
	Female	*Reference*						
Age in years		0.28 (0.67)	(−1.05 to 1.62)	0.04	0.4	0.677		
Mother’s employment status	Unemployed	5.10 (2.38)	(0.36 to 9.84)	0.19	2.1	*0.035*		
	Employed	*Reference*						
Experiences of childhood adversity	Yes	4.43 (1.74)	(0.97 to 7.88)	0.25	2.5	*0.013*		
	No	*Reference*						
Experienced sexual/physical violence	Yes	2.44 (3.23)	(−3.99 to 8.87)	0.07	0.8	0.452		
	No	*Reference*						
R^2^							0.391	

Dependent variable total VAS score

*Beta* unstandardized coefficient, *SE* standard error, *β* standardized coefficient

Italicized numerals are strong positive correlations

** Correlation is significant at the 0.01 level (two-tailed)

When individual predictors using standardized beta scores were examined, emotional symptoms and experiencing adversity in childhood explained the most variance in the attachment insecurity, followed by mother’s employment status. Controlling for all other predictors adolescents who had higher emotional scores, had experienced childhood adversity, whose mothers were unemployed had 0.25, 0.25 and 0.19 higher attachment insecurity scores respectively than those with lower emotional scores, those who had not experienced childhood adversity, and those whose mothers were employed.

## Discussion

In our sample, a relatively high number (89.9%) of adolescents had overall high vulnerable attachment security scores. Additionally, we found that the adolescents from the low SES school had higher vulnerable attachment scores than those from the middle SES school. This finding is akin to results from a study among adolescents in the US that showed that poverty status may decrease one’s security to attachment figures [16]. Financial hardship may negatively impact child and adolescent development as it typically affects both the adolescent and the caregiver, leaving the adolescent increasingly in need of support and comfort from primary attachment figures at a time when these figures are most stressed and least able to provide this support. Such situations are likely to lead to attachment insecurity [16].

Adolescents’ experiences of adversity as well as sexual or physical abuse were associated with increased vulnerable attachment towards parents/caregivers. These results were similar to the work of Sternberg et al. [25], who found that recent abuse and perpetrator status predicted adolescents’ attachments to their mothers. Those individuals who were exposed and themselves abused would have even more negative perceptions of their attachments. Research has also shown that early experiences of adversity (e.g. childhood abuse), the lack of childhood care, and the lack of affective bonds with a caring adult, constitute a greater risk factor of emotional problems than even the death of a parent [26]. This suggests that if difficulties accumulate over time, they may trigger symptoms of emotional problems in vulnerable adolescents. Early childhood adversities, including parental separation or neglect, may affect children negatively, to a point where they experience difficulty in building a stable and positive relationship with a parent, thus increasing family discord and contributing to inadequate parenting styles and practices.

We found a significant positive relationship between increased emotional and behavioral problems and increased vulnerable attachment. Secure attachment, especially during adolescence, may serve as a buffering system in the developmental stage of many internal and external pressures. A study conducted in two informal settlements in Nairobi, for example, found that parental monitoring moderated the association between adverse childhood events and delinquent behavior [27], which suggests that close ties between parents and children may allow for greater self-expression and enable parents to provide better care for their children. A longitudinal study of 160 early-adopted children from infancy to adolescence, found that supportive and sensitive parenting in adolescence, may protect adolescents from developing inhibited behavior and internalizing behavior problems [28]. A Dutch study focusing on a similar population of youth, found that unfavorable parent-adolescent attachment at baseline was related to increased risk of mental health problems at follow up [29]. Secure attachment can therefore be said to enhance the individuals coping abilities, which they use to assess potentially stressful situations while evaluating their resources (e.g. parent-adolescent relationship) to handle the situation [29].

In summary, attachment insecurity was found to influence the development of emotional and behavioral problems in adolescents. The more insecurely attached a child is, the more vulnerable she or he may be to develop emotional and behavioral problems. This is consistent with literature associating attachment insecurity with internalizing and externalizing behaviors at several points in a child’s lifespan [30, 31]. Overall, these findings suggest that in adolescence, security in attachment organization is not simply a marker of one’s relationship with parents, but there are various psychosocial factors that influence one’s attachment security.

Our study was not without its own limitations. The first limitation emanated from our sample that was restricted to adolescents in schools in an urban area; therefore, the results are not necessarily generalizable to a larger Kenyan population. Second, conducting attachment research in a Kenyan population sample was challenging because of the lack of locally-validated measures. The attachment measure used may have had some limitations, for example we were not able to take into account different aspects of the attachment relationship that are unique to the African ethno-cultural setting. The cross-sectional design of the study is also a potential limitation in that the time order of variables is not known and ongoing distress could have biased attachment scores. Finally, a comprehensive study of child and adolescent attachment requires a bifocal assessment, requiring some interviewing with the parents, which was outside the scope of this study.

## Conclusions

In tandem with other studies, our study lends support to the notion that attachment security influences the adolescent’s susceptibility to develop emotional and behavioral problems. Further research with larger and more representative samples is needed to explore the different factors that can be considered risk or protective to the development of emotional, attachment and behavioral problems in adolescents.

Past research results as well as our findings, further suggest, need to develop programs aimed at sensitizing parents on the importance of parental roles and parent child attachment so as to mitigate development of psychopathology in children and adolescents. The development of school mental health programs that can address emotional and behavioral needs of the pupils as well as encourage early screening for those in high-risk families for psychological disorders in the Kenyan context may be imperative measures to improve child and adolescent well-being.
